# Adult T-cell leukemia/lymphoma following elevation of serum levels of soluble cytokine receptors, sCD25 and sCD30

**DOI:** 10.1186/1742-4690-11-S1-P1

**Published:** 2014-01-07

**Authors:** Shigeki Takemoto, Koji Uzawa, Kazuki Morita, Ratiorn Pornkuna, Yoshio Haga, Masako Iwanaga, Yasuko Sagara, Fumio Kawano, Toshiki Watanabe

**Affiliations:** 1 Clinical Research Institute, National Hospital Organization Kumamoto Medical Center, Kumamoto, Japan; 2 Department of Hematology, National Hospital Organization Kumamoto Medical Center, Kumamoto, Japan; 3 Department of International Medical Cooperation, Graduate School of Medical Sciences Kumamoto University, Kumamoto, Japan; 4 Research Laboratories, KYOWA MEDEX CO., LTD., Shizuoka, Japan; 5 Joint Study on Predisposing Factors of ATL Development (JSPFAD), Tokyo, Japan; 6 School of Public Health, Teikyo University, Tokyo, Japan; 7 Department of Quality, Japanese Red Cross Kyushu Block Blood Center, Fukuoka, Japan; 8 Department of Medical Genome Science, Graduate School of Frontier sciences, The University of Tokyo, Tokyo, Japan

## 

Adult T-cell leukemia /lymphoma (ATL) caused by human retrovirus, HTLV-1, is a neoplasm of mature T-cell. ATL is still an incurable disease with approximately 1,000 annual deaths in Japan. The useful biomarkers are required to achieve the success of treatment. In order to test whether or not two soluble cytokine receptors (sCD25 and sCD30) are useful biomarkers of the development of ATL, we investigated changes in the concentration of the two markers in 11 HTLV-1 carriers (pre/post ATL progression) and six patients with chronic ATL (pre/post acute crisis). Serum (or plasma) samples of newly diagnosed or referred ATL patients and HTLV-1 carriers were preserved in freezer to measure the level of soluble proteins, sCD25 and sCD30, using ELISA. The sCD25 concentration was measured by Cell freeN IL-2R (Kyowa Medex, Japan) and Determiner CL IL-2R (Kyowa Medex, Japan). The sCD30 concentration was measured by Human sCD30 Platinum ELISA (eBioscience). The probability of ATL development from HTLV-1 carrier state was analysed by Kaplan-Meier method. We found that the elevation of soluble cytokine receptors was one of the earlier events of ATL development. In addition, chronic type patients followed in our hospital showed sCD30 as well as sCD25 elevation prior to the diagnosis of acute crisis. Our findings suggest that a combination of sCD25 and sCD30 facilitates accurate diagnosis and intervention of ATL as useful biological markers. Further careful follow-up should be required for HTLV-1 carriers with elevated level of cytokine receptors, sCD25 and sCD30 to diagnose the early phase of ATL.

